# Porcine Astrovirus 4 Detection in Lesions of Epitheliotropic Viral Infection in the Porcine Respiratory Tract

**DOI:** 10.1155/2023/9113355

**Published:** 2023-04-15

**Authors:** Michael C. Rahe, Alyona Michael, Pablo Enrique Piñeyro, Jennifer Groeltz-Thrush, Rachel J. Derscheid

**Affiliations:** Iowa State University, Ames, Iowa, USA

## Abstract

Astroviruses infect mammals and birds resulting in either gastroenteritis, neurologic disease, or asymptomatic infection. Porcine astrovirus 4 (PoAstV4) has previously been detected in the upper respiratory tract of pigs with clinical respiratory disease; however, proof of respiratory tract infection and association of the virus with respiratory pathology have not been shown. In this retrospective study of young pigs with clinical respiratory disease of unknown etiology, PoAstV4 was detected with RNA *in situ* hybridization in lesions consistent with epitheliotropic viral infection in 85 of 117 pigs. This is the first report associating an astrovirus with respiratory pathology.

## 1. Introduction

Astroviruses are nonenveloped, positive-sense, single-stranded RNA viruses that have primarily been associated with enteric disease or asymptomatic infection in numerous species, including humans [1]. In the pig, there are currently five known lineages of astroviruses [2]. Porcine astrovirus 3 causes polioencephalomyelitis while porcine astrovirus 5 has been associated with atrophic enteritis [3–5]. Porcine astrovirus-4 (PoAstV4) has previously been detected in feces from pigs with diarrhea and in nasal swabs from pigs with clinical respiratory disease [6, 7]. However, identification of the agent within microscopic lesions in either the intestines or respiratory tract has not been demonstrated.

Over the last three years at the Iowa State University Veterinary Diagnostic Laboratory (ISU VDL), there have been multiple cases of acute to subacute tracheitis and/or bronchitis where PCR testing was negative for influenza A virus (IAV) and other reported causes of epitheliotropic viral infection. Subsequent next generation sequencing (NGS) of affected lungs from these cases revealed numerous reads of porcine astrovirus 4. Therefore, we hypothesize that PoAstV4 is associated with lesions of epitheliotropic viral infection. A retrospective analysis of cases of porcine respiratory disease submitted to the ISU VDL from January 1, 2019 to August 1, 2022 was conducted to evaluate the presence of PoAstV4 within microscopic lesions consistent with epitheliotropic viral infection of airways.

## 2. Materials and Methods

### 2.1. Case Selection

**Figure 1:**
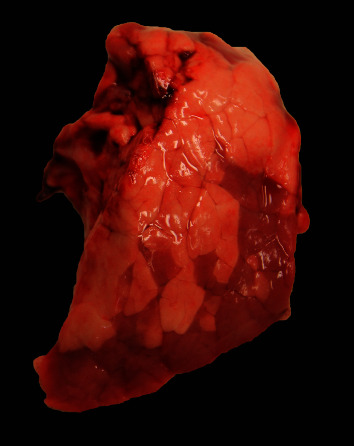
Macroscopic lesion of multifocal to coalescing lobular atelectasis of a cranioventral lung lobe from a PoAstV4-positive case. Affected areas are darker pink to red, depressed, and have distinct lobular margins. This is indistinguishable from the common gross findings observed in acute IAV infections.

**Figure 2:**
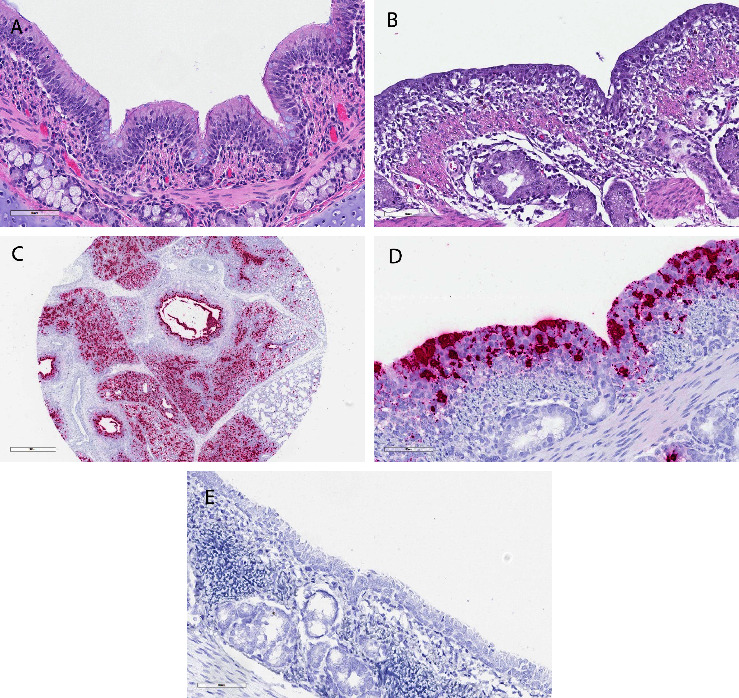
Microscopic lung lesions with direct detection of PoAstV4. (a) (300x, H&E) normal bronchus, 4-week-old pig. There are normal ciliated, pseudostratified columnar epithelium with moderate numbers of goblet cells. There are minimal resident lymphocytes in lamina propria and few migrating through the epithelium. Normal submucosal glands are presented subjacent to the muscularis mucosae. (b) (300x, H&E) representative microscopic findings of bronchitis. Respiratory epithelium is attenuated, vacuolated, and hyperplastic with infiltration of the lamina propria by numerous lymphocytes, plasma cells, and rare eosinophils which also cuff submucosal glands. (c) (25x, RNAscope) a single punch biopsy with positive signal. PoAstV4 direct detection (red) is apparent in airway epithelium and within respiratory parenchyma. (d) (300x, RNAscope) PoAstV4 direct detection (red) in affected respiratory epithelium as well as in epithelium of submucosal glands by RNA *in situ* hybridization. (e) (300x, DapB RNAscope) this RNAscope probe slide targets the DapB gene from the Bacillus subtilis strain SMY. This shows no false positive host RNA detection. Scale bars for images a, b, d, and e are 80 *μ*m and 900 *μ*m for image c.

From January 2019 to August 2022, a total of 1,120 cases of either tracheitis and/or bronchitis of unknown etiology were diagnosed at the ISU VDL. For retrospective analysis, age parameters were set at neonate to 6 weeks of age (42 days), as diagnostician experience had suggested that this age group was the most likely to be affected by PoAstV4. This removed 587 cases from analysis. Following evaluation of diagnostic reports, an additional 326 cases were removed primarily due to the lack of influenza A virus (IAV) PCR testing. Only IAV negative cases were included in the retrospective analysis, as IAV is highly prevalent in the US swine herd and, to date, is the most frequent cause of bronchitis and/or tracheitis in pigs [8]. Evaluation of histopathology lesions and confirmation of the availability of formalin-fixed paraffin-embedded (FFPE) tissue blocks was completed on the remaining 207 cases. This resulted in the elimination of 90 cases due to either (1) chronic lesions of tracheitis or bronchitis, characterized by normal respiratory epithelium with lymphoplasmacytic infiltration of the lamina propria and/or submucosa or (2) no FFPE tissue remaining in the block for additional testing. Chronic cases were removed as primary infectious virus was likely no longer be present in lesions. One hundred and seventeen cases were confirmed to contain at least one of the following: respiratory epithelial necrosis, attenuation, or squamous metaplasia in either the trachea and/or airways (bronchi or bronchioles) of the lung (Figure 1 and 2). Additionally, corresponding FFPE tissue blocks were available for these cases.

### 2.2. Tissue Microarray Creation

Pulmonary tissue microarrays containing six unique cases and a positive control per slide were created by using a 5.0 mm diameter punch biopsy to excise formalin-fixed tissues with representative lesioned regions of interest from the original FFPE tissue blocks. For each case, a trachea with lesions was selected preferentially to affected bronchi or bronchioles for microarray production, and if no trachea was present, a bronchus or bronchi with lesions were prioritized over affected bronchioles. In total, fixed trachea was represented in 19 cases, and 18 of these were preferentially selected for analysis due to the presence of lesions consistent with epitheliotropic viral infection. The remaining 99 tissue punches were taken from lung sections that contained affected airways (bronchi and bronchioles). All but three cases had bronchi included in the tissue sampled. These three cases had bronchiolitis with no evidence of bronchitis in the evaluated sections.

### 2.3. PoAstV4 RNA *In Situ* Hybridization and Histopathologic Evaluation

PoAstV4 RNA *in situ* hybridization (ISH) targeting ORF-1a (RNAscope; TriStar Technology LLC, https://tristargroup.us) was run on all twenty tissue microarray slides. Three diagnostic pathologists independently evaluated all PoAstV4 RNAscope slides for the presence of PoAstV4 RNA signal in airway epithelium with a two-thirds majority used to resolve any discordance in results.

## 3. Results and Discussion

PoAstV4 RNA was detected in affected respiratory epithelium in 85 (73%) of 117 evaluated cases (Table 1 and Figure 2). Of the 18 evaluated tracheas, 13 (72%) had detection of PoAstV4 RNA. 72 (73%) of 99 cases had detection of PoAstV4 RNA in bronchial and/or bronchiolar respiratory epithelium.

Clinical case data were not available for all cases. The 85 cases where PoAstV4 was detected in respiratory epithelium were submitted from a total of 15 states (Table 2). The median and average age of pigs with PoAstV4 detection was 21 and 23.2 days of age, respectively. Respiratory morbidity was indicated as a primary concern by the referring veterinarian in 80 (94%) out of 85 PoAstV4-positive submissions, with coughing described in 63 (79%) out of 89 submissions that reported specific clinical signs. Porcine reproductive and respiratory syndrome virus (PRRSV), which causes an interstitial pneumonia and is one of the leading causes of respiratory disease in pigs, was tested for via PCR in 62 (73%) of the 85 cases with positive detection of the virus in 10 (16%) of 62 cases. Interstitial pneumonia was diagnosed by the pathologist in six of these ten cases; that is, only 6 of the 85 cases (7%) with lesions and detection of PoAstV4 had concurrent diagnosis of PRRSV. Porcine parainfluenza virus-1, a paramyxovirus which has previously been associated with mild rhinitis and mild tracheitis in young pigs, was tested for via PCR in 30 (35%) of 85 cases [9] with all cases negative. Bacterial bronchopneumonia, of varying severities, was confirmed by histopathology in 54 (64%) of 85 cases where PoAstV4 was detected in respiratory epithelium.

The wide geographic distribution and detection of this agent in cases from over a three-year period suggests that this virus has been circulating for some time and is likely endemic in commercial swine in the US. The distribution and abundance of this virus makes coinfections between PoAstV4 and IAV likely. Future investigation of PoAstV4 and IAV coinfection would be of interest to evaluate potential clinical potentiation or synergism.

The PoAstV4 sequences from Padmanabhan and Hause [6] showed a high degree of genetic diversity compared to other previously reported sequences of PoAstV4. In another study of PoAstV4 in swine feces of diarrheic pigs, genetic variability of 8 strains of PAstV4 ranged from 63.6 to 99.7% [10]. This suggests substantial genetic diversity within the PoAstV4 lineage and possible resulting differences in tissue tropism. Further work is necessary to understand the breadth of genetic diversity, sites of mutagenesis within the genome, and potential differences in pathogenicity based on sequence differences.

The sheer volume of cases from the ISU VDL with diagnoses of tracheitis and/or bronchitis (>1,000) over the defined study period required limiting our evaluation to pigs under 6 weeks of age, the demographic of highest empirical susceptibility, based on our diagnostic experience. However, infection and resulting disease in older animals appear likely and worthy of further investigation. While isolation of porcine astroviruses has proven difficult, Tao et al. [7] reports the isolation of PoAstV4 using PK15 cells, but this has yet to be reported by additional researchers.

PoAstV4 has previously been detected in nasal and fecal swabs collected from suckling piglets with unexplained respiratory disease [6]. However, detection does not equal causation, as infectious agents that are shed in feces can easily be inhaled by animals that keep their nose close to the ground, such as the pig, resulting in detection of an agent in the upper respiratory tract without true infection of the respiratory tract. Here, we take the next step towards proving PoAstV4 is a cause of respiratory disease in pigs by identifying the virus within epithelial cells in microscopic lesions of epitheliotropic viral infection in the trachea, bronchi, and bronchioles. The association between virus and lesion is particularly strong in this study although association does not equal causation either, and the fulfillment of Koch's postulates should be the final step in proving that this virus is a cause of respiratory disease in pigs.

## Figures and Tables

**Table 1:** Summary of PoAstV4 RNA detection in lesions of epitheliotropic viral infection in neonatal to six-week-old pigs.

	PoAstV4 (+) cases/total cases evaluated
PoAstV4 RNA detected in epitheliotropic lesions in respiratory tract	85/117 (73%)
PoAstV4 RNA detected in affected tracheal epithelium	13/18 (72%)
PoAstV4 RNA detected in affected bronchus or bronchiolar epithelium	72/99 (73%)

**Table 2:** Distribution and proportion of positive PoAstV4 cases in the US. For comparison, confirmed positive IAV cases with histopathological lesions in pigs <6 weeks old, from the same time frame as PoAstV4-positive cases.

States	PoAstV4 (+) cases	IAV diagnoses
Iowa	40 (47%)	922 (50%)
South Dakota	8 (9%)	38 (2%)
Illinois	8 (9%)	168 (9%)
North Carolina	8 (9%)	110 (6%)
Missouri	4 (5%)	94 (5%)
Indiana	4 (5%)	87 (5%)
Virginia	3 (4%)	35 (2%)
Nebraska	2 (2%)	53 (3%)
Ohio	2 (2%)	53 (3%)
Arkansas	1 (1%)	42 (2%)
Kentucky	1 (1%)	6 (<1%)
Minnesota	1 (1%)	100 (6%)
Oklahoma	1 (1%)	8 (<1%)
Tennessee	1 (1%)	1 (<1%)
Wisconsin	1 (1%)	28 (2%)
Pennsylvania	0 (0%)	43 (2%)
Arizona, California, Colorado, Kansas, Michigan, Utah	0 (0%)	44 (2%)
Total	85 cases (100%)	1832 cases (100%)

## Data Availability

The data supporting the results reported in this study are available upon request from the corresponding author (Rahe).
